# A Real-Time Mature Hawthorn Detection Network Based on Lightweight Hybrid Convolutions for Harvesting Robots

**DOI:** 10.3390/s25165094

**Published:** 2025-08-16

**Authors:** Baojian Ma, Bangbang Chen, Xuan Li, Liqiang Wang, Dongyun Wang

**Affiliations:** 1 School of Mechatronic Engineering, Xinjiang Institute of Technology, Aksu 843100, China; 11813019@zju.edu.cn (B.M.); 2018017@xjit.edu.cn (X.L.); 2019049@xjit.edu.cn (L.W.); 2 School of Mechatronic Engineering, Xi’an Technological University, Xi’an 710021, China; 3 College of Engineering, Zhejiang Normal University, Jinhua 321004, China; zsdwdy@zjnu.edu.cn

**Keywords:** complex environment, harvesting robot, real-time detection, lightweight, deployment application

## Abstract

Accurate real-time detection of hawthorn by vision systems is a fundamental prerequisite for automated harvesting. This study addresses the challenges in hawthorn orchards—including target overlap, leaf occlusion, and environmental variations—which lead to compromised detection accuracy, high computational resource demands, and poor real-time performance in existing methods. To overcome these limitations, we propose YOLO-DCL (group shuffling convolution and coordinate attention integrated with a lightweight head based on YOLOv8n), a novel lightweight hawthorn detection model. The backbone network employs dynamic group shuffling convolution (DGCST) for efficient and effective feature extraction. Within the neck network, coordinate attention (CA) is integrated into the feature pyramid network (FPN), forming an enhanced multi-scale feature pyramid network (HSPFN); this integration further optimizes the C2f structure. The detection head is designed utilizing shared convolution and batch normalization to streamline computation. Additionally, the PIoUv2 (powerful intersection over union version 2) loss function is introduced to significantly reduce model complexity. Experimental validation demonstrates that YOLO-DCL achieves a precision of 91.6%, recall of 90.1%, and mean average precision (mAP) of 95.6%, while simultaneously reducing the model size to 2.46 MB with only 1.2 million parameters and 4.8 GFLOPs computational cost. To rigorously assess real-world applicability, we developed and deployed a detection system based on the PySide6 framework on an NVIDIA Jetson Xavier NX edge device. Field testing validated the model’s robustness, high accuracy, and real-time performance, confirming its suitability for integration into harvesting robots operating in practical orchard environments.

## 1. Introduction

Crataegus pinnatifida Bge, a member of the Rosaceae family, is one of China’s important medicinal and edible plants, with a long history of cultivation and consumption. Historical records indicate that hawthorn has been used in China for over 2000 years. It is renowned for its significant health benefits, including promoting digestion, invigorating blood circulation, and preventing cardiovascular diseases. As such, it is widely utilized in food processing and traditional Chinese medicine formulations [1,2]. With the growing emphasis on health-conscious consumption, the demand for hawthorn products has steadily increased, driving the expansion of its cultivation area and yield. According to statistics, by 2025, the domestic hawthorn planting area is projected to reach 86,700 hectares, with an annual production exceeding 1.5 million tons. The primary cultivation areas are located in Shandong, Shanxi, Henan, and Hebei provinces, with each province having a planting area exceeding 10,000 hectares. However, this industry faces a critical bottleneck: harvesting remains predominantly reliant on manual labor. This dependence leads to high labor intensity, low efficiency, and escalating human costs, severely constraining the development of the hawthorn industry and the advancement of smart agriculture. Consequently, developing automated harvesting technology is imperative to overcome this bottleneck and propel the intelligent upgrading of the industry.

To address this challenge, researchers have explored automated solutions. Zhai Yukun et al. [3] proposed a mechanical arm-based hawthorn-picking mechanism that attempted to partially replace human labor. Although this approach partially reduces manual effort, it crucially still requires manual visual guidance and control, falling short of achieving full automation. Therefore, realizing truly autonomous harvesting necessitates robust machine vision for fruit recognition. As artificial intelligence continues to integrate with agricultural equipment, automatic recognition and picking technologies based on object detection are considered essential for achieving the automation of fruit tree harvesting [4]. Nevertheless, achieving high-precision, real-time recognition of hawthorn fruits presents significant difficulties due to their inherent growth characteristics and complex orchard environments. Hawthorn fruits typically grow densely and are frequently obscured by leaves and branches, compounded by variations in lighting conditions. Thus, tackling these vision challenges is paramount and constitutes a core technical hurdle in automated hawthorn harvesting.

In recent years, machine vision technology has become a research hotspot in fruit recognition and ripeness judgment. Although research specifically on hawthorn-picking robots is still in its infancy, extensive studies exist for other fruits like apples, bananas, grapes, watermelons, and tomatoes, employing both traditional image processing and deep learning-based methods. Traditional image processing techniques primarily rely on low-level image features such as color, texture, and shape, and employ color space transformation and image segmentation algorithms to separate the fruit from the background [5]. For instance, Mo Songtao et al. [6] constructed a banana ripeness judgment model based on genetic algorithms and support vector machines (SVM), achieving an average recognition accuracy of 86.2% by manually extracting fruit cross-sectional angle features. Additionally, Zou Wei [7] used RGB to HSV space conversion and combined histogram peak values from the H channel to classify citrus ripeness, achieving an accuracy of over 90%. While demonstrating practical value in specific contexts, these traditional methods exhibit inherent limitations: they often suffer from slow detection speeds, constrained feature representation capabilities, and notably poor adaptability to complex scenarios involving occlusion, overlap, and varying lighting conditions. Critically, these limitations are severely exacerbated when applied to the challenging case of hawthorn recognition, where targets are characteristically small, densely clustered, and heavily occluded—frequently resulting in missed detections and reduced accuracy.

In contrast, deep learning-based object detection algorithms, renowned for their superior feature extraction and representation capabilities, have demonstrated significantly enhanced adaptability and accuracy in fruit recognition tasks [8]. These methods typically employ convolutional neural networks (CNNs) to extract multi-scale features from images, capturing both shallow color information and deep semantic information. Consequently, algorithms such as Fast R-CNN [9], SSD [10], and the YOLO series [11] have been widely applied in fields such as for fruit ripeness recognition, yield estimation, and harvesting path planning. Numerous advancements have been made to boost performance: Li Li et al. [12] proposed a multi-channel information fusion model based on YOLOv5 for citrus fruit recognition in complex environments. By integrating the CBAM attention mechanism and the SIoU loss function optimization strategy, their model achieved an average precision of 91.1%. Moreover, Gu Hongyu et al. [13] optimized the YOLOv5 structure by introducing the information aggregation-distribution module and the WIoU loss function, effectively improving the accuracy of seed tuber bud-eye detection and enhancing the model’s mAP value to 90.1%. To address efficiency and model size, Luo Zhichong et al. [14] developed a lightweight YOLOv8s-based model for passion fruit using knowledge distillation and pruning, increasing FPS to 13.87 and reducing memory by 62.10%, though its mAP remained below 90% under occlusion. Directly tackling occlusion and overlap, Dong Genggeng et al. [15] addressed fruit overlap and leaf occlusion issues by integrating the CA attention mechanism into YOLOv5s, achieving a detection accuracy of 93.4%. Furthermore, common strategies to mitigate failures caused by small targets and occlusion include data augmentation. For example, Song Huaibo et al. [16] introduced the mosaic data augmentation technique during the training phase, effectively enhancing the model’s ability to detect small-scale targets. Yang et al. [17] introduced a residual grid structure into YOLOv8 to improve the fusion of multi-scale features in strawberry images. Wu et al. [18] optimized the loss function to improve fruit detection integrity, while Zheng et al. [19] applied an attention mechanism to enhance the target feature extraction for recognizing Chinese jujube under occlusion. Despite these significant strides in improving general fruit detection accuracy, formidable challenges persist when confronting scenarios characterized by exceptionally small target size, extreme density, and severe occlusion—precisely the defining characteristics of hawthorn fruits in natural orchards. Moreover, research specifically targeting hawthorn recognition under these demanding conditions remains relatively scarce, underscoring the need for more effective solutions tailored to this unique application.

To overcome these persistent detection challenges for small, dense, and heavily occluded hawthorn fruits in complex orchard environments, this study proposes YOLO-DCL, a high-precision real-time detector based on YOLOv8n. Key innovations include: (1) A dynamic group convolution shuffle transformer (DGCST) module in the backbone enhancing feature extraction for small/occluded targets; (2) A hierarchical scale-aware pyramid feature network (HSPFN) integrating coordinate attention (CA) and refined C2f in the neck to improve multi-scale fusion for overlap/size variation; (3) A lightweight Detect_LIH head optimizing memory/speed; (4) PIoUv2 loss replacing CIoU for superior bounding box regression and faster convergence. The model targets robust automated harvesting systems and will be validated on an NVIDIA Jetson Xavier NX platform with a PySide6 framework under challenging field conditions (e.g., backlight, heavy occlusion).

This paper is organized as follows: Section 2 introduces the foundational materials and methodologies underpinning the study, including a systematic description of the technical framework for hawthorn fruit identification. Section 3 delineates the experimental design, specifies the performance assessment criteria, and synthesizes the key outcomes derived from empirical validation. The Section 4 articulates overarching conclusions and proposes actionable avenues for hawthorn automated harvesting.

## 2. Materials and Methods

### 2.1. Construction of Hawthorn Image Dataset in Complex Environments

#### 2.1.1. Image Dataset Collection

**Figure 1:**
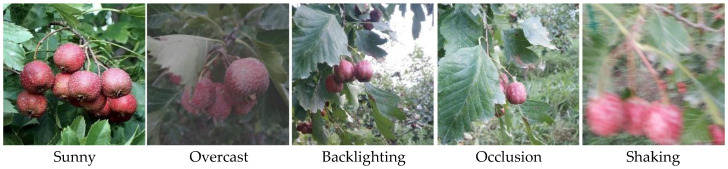
Example of collected image data.

To study hawthorn growth under representative cultivated conditions, this study selected a hawthorn plantation in the Aksu region of Xinjiang. The plantation uses a dwarfing, high-density planting pattern, which facilitates accurate fruit detection from both front and lower perspectives by the harvesting robot via a depth-sensing camera, while the low canopy height provides ample workspace for the robotic arm. During data collection, diversity and comprehensiveness were prioritized across varying lighting conditions (backlighting, front lighting), camera angles (overhead, level, upward), and distances (0.1–1.0 m). Primary equipment included a Huawei Nova 7 smartphone (Huawei Technologies Co., Ltd., Shenzhen, China) and an Intel RealSense D456 depth camera (Intel Corporation, Santa Clara, CA, USA). Data were collected from 10 to 20 September 2024, yielding 1454 hawthorn images in JPEG format. Examples are shown in Figure 1.

#### 2.1.2. Image Dataset Augmentation and Processing

**Figure 2:**
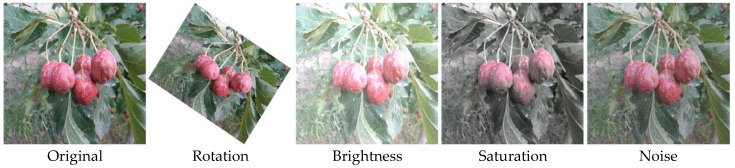
Examples of image data augmentation.

To enhance the robustness and generalization ability of the hawthorn detection model in natural environments and to accurately extract hawthorn growth posture features under various complex conditions, this study employed a data augmentation strategy based on the orchard planting pattern and the harvesting robot’s operational mode. Random rotations, brightness adjustments, saturation variations, and noise addition were applied using Python to the collected raw data. These operations aimed to simulate complex environmental factors that the robot might encounter during harvesting, such as lighting changes, dust interference, and mechanical vibrations. The raw data, augmented through these methods, resulted in a final dataset of 1859 image samples. Examples of the augmented samples are shown in Figure 2. The dataset was split into training (1302 images), validation (372 images), and testing (185 images) sets in a 7:2:1 ratio, ensuring no overlap between the sets. During the construction of the hawthorn dataset, the PASCAL VOC format was followed, and labeling was performed using LabelImg software. Only harvestable hawthorn fruits were annotated, labeled as the “Hawthorn” category; non-harvestable fruits were left unlabeled.

### 2.2. The YOLO-DCL Hawthorn Detection Model Based on Lightweight Convolution

**Figure 3:**
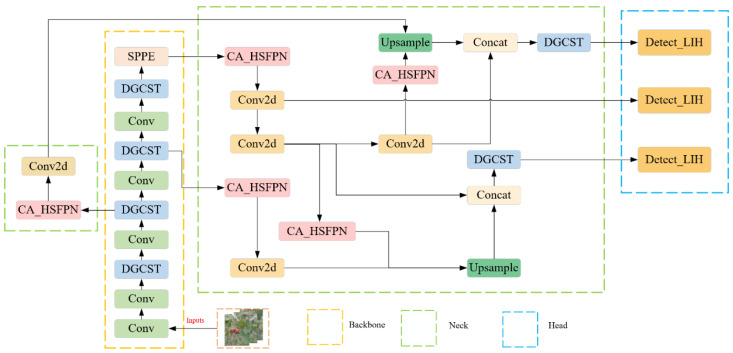
YOLO-DLC network structure.

For real-time and precise hawthorn detection in complex orchard natural environments, deployable on low-power edge devices, a model requiring high speed and accuracy is essential. This study selects the current mainstream YOLOv8n as the baseline model. Its architecture consists of backbone, neck, and head networks and represents the smallest model within the YOLOv8 series in terms of memory footprint. Although YOLOv8n offers good real-time performance, its deployment on edge devices is hampered by challenges such as missed detections, reduced accuracy, and a relatively large model size, particularly in complex orchard settings. To address these limitations, we proposed an improved lightweight real-time object detection model named YOLO-DCL, whose overall architecture is depicted in Figure 3. The main innovations include the following four aspects:

(1) Replacing the original C2f module in YOLOv8n’s backbone network with a dynamic group convolution shuffle transformer (DGCST) module to improve feature extraction, especially in complex backgrounds.
(2) In the neck network, incorporating the coordinate attention (CA) mechanism into the feature pyramid network (FPN) to construct a hierarchical scale-aware pyramid feature network (HSPFN), which improves the C2f structure.
(3) Designing a new lightweight detection head, Detect_LIH, based on shared convolution and batch normalization to significantly reduce model parameters and computational load while maintaining detection performance.
(4) Replacing the default CIoU loss function with the PIoUv2 loss function to improve detection accuracy and accelerate model convergence, optimizing the bounding box regression process more effectively.

#### 2.2.1. Dynamic Group Convolution Shuffle Transformer Module

**Figure 4:**
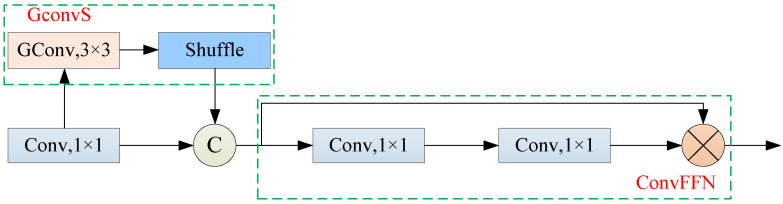
DGCST network structure.

The C2f module in YOLOv8n’s backbone network [20] enhances the model’s non-linearity and representational ability by increasing the computational complexity and memory usage to improve feature extraction. However, in applications such as resource-constrained mobile terminals, real-time detection tasks, and lightweight orchard harvesting robots, the C2f module faces significant bottlenecks in computational efficiency and deployment performance. To address these issues, this study proposed replacing the C2f module with an improved dynamic group convolution shuffle transformer (DGCST) module [21,22,23]. This module integrates the vision transformer (ViT) structure, group convolution, and channel shuffle mechanisms. By improving feature representation and inter-channel information interaction efficiency; the DGCST effectively reduces the parameter scale and computational complexity. Specifically, DGCST retains the advantage of cross-channel feature interaction, introduces the transformer structure to capture richer long-range dependencies, and enhances the model’s robustness and discriminative ability in complex orchard scenes. Its structure is shown in Figure 4.

As shown in Figure 4, the core design of the DGCST module is based on a 3:1 division strategy, where a quarter of the feature channels undergo group convolution and channel shuffle operations. The specific operation mechanism is as follows: first, dynamic group convolution is applied, introducing a small feature selector for each group, and the number of convolutional kernel groups is adaptively chosen according to the feature strength, which helps better capture features while reducing the number of computation parameters. The computational operations can be summarized by Formulas (1) and (2):

(1) $$x^{\prime }=[\hat{f}(x^{1}),\hat{f}(x^{2}),\ldots \ldots ,\hat{f}(x^{N})]$$

(2) $$\hat{f}(x^{i})=f(x^{i},\theta^{i})\cdot \kappa (x^{i},\phi^{i})$$

In the formulas, $x\in R^{C\times H\times W}$ and $x^{\prime }\in R^{C^{\prime }\times H^{\prime }\times W^{\prime }}$ represent the input and output of the current layer, $x^{i}\subseteq x$ is the subset of input channels divided into the (i)-th group, *N* is the number of groups, *f* is the parameter θ that performs conventional convolution, κ is the prediction function, and ϕ is the parameter of κ.

Dynamic group convolution is a channel-sparse connection method that limits the efficiency of information flow between channels to some extent. Therefore, after performing dynamic group convolution, a channel shuffle operation is applied. This operation allows the group convolution, originally composed of (*g*) groups, to output *g* × *n* channels. Then, by reshaping the output channels to (*g*, *n*) and transposing them, the next layer’s input is formed. The channel shuffle operation reorganizes the features from the hawthorn feature map, ensuring that the convolution operations in the next layer receive inputs from different groups, thus increasing the diversity of hawthorn features without increasing computational complexity. Based on this, the part after group convolution and channel shuffle is connected with other branches and fed into the fully connected feed-forward network (ConvFFN) in the transformer architecture. This design not only effectively reduces computational demands but also better matches the characteristics of convolutional neural networks. Additionally, to further optimize model performance, the number of detection heads is reduced from the original three to two, which not only reduces the model’s computational burden but also improves detection efficiency. Overall, replacing the C2f module with the DGCST module significantly reduces the model’s parameter count and computation load, avoids overfitting, and enhances real-time detection capability and adaptability in complex hawthorn environments while maintaining model robustness and generalization ability.

#### 2.2.2. Hierarchical Scale Feature Pyramid Network (HSFPN)

**Figure 5:**
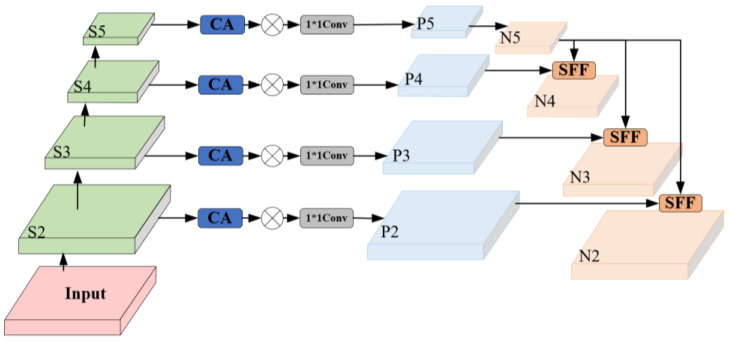
HSFPN network structure.

To further enhance the neck network’s ability in multi-scale feature extraction and fusion, and to comprehensively improve object detection accuracy and inference speed, this study introduced a secondary improvement in the multi-level scale feature pyramid network, HSFPN [24,25]. This module consists of two parts: the feature selection module and the feature fusion module, as shown in Figure 5.

In the feature selection module, feature maps from different scales are first filtered and integrated through a selective feature fusion (SFF) mechanism. Specifically, the fusion strategy focuses on the collaborative modeling of high-level semantic information and low-level positional information to achieve efficient information sharing across feature layers. In this module, the coordinate attention mechanism (CA) replaces the channel attention mechanism. The CA mechanism utilizes bidirectional position encoding to achieve collaborative optimization of cross-channel information modeling and spatial positioning ability in lightweight networks. In the coordinate embedding, the CA attention mechanism uses pooling kernels of sizes (*H*, 1) and (1, *W*) to encode each channel along the horizontal and vertical coordinates, respectively. The output at height (*h*) in the (*c*)-th channel is expressed by Formula (3), and the output at width (*w*) in the (*c*)-th channel is expressed by Formula (4). In the coordinate attention generation, the position information from the horizontal and vertical directions is concatenated to form a 1 × 1 convolution transformation function, with the operation described in Formula (5). By applying the convolution operation, the attention weights (*g^h^*) and (*g^w^*) are calculated, as expressed in Formulas (6) and (7). The final coordinate attention is computed by multiplying the attention weights, as shown in Formula (8):

(3) $$z_{c}^{h}(h)=\frac{1}{W}\sum_{0\leq i<w}x_{c}(h,j)$$

(4) $$z_{c}^{w}(w)=\frac{1}{H}\sum_{0\leq j<H}x_{c}(i,w)$$

(5) $$f=\delta (F_{1}([z^{h},z^{w}]))$$

(6) $$g^{h}=\sigma (F_{h}(f^{h}))$$

(7) $$g^{w}=\sigma (F_{w}(f^{w}))$$

(8) $$y_{n}(i,j)=x_{n}(i,j)\times g_{n}^{h}(i)\times g_{n}^{w}(j)$$

In the formula, *H* is the height of the input feature map, *W* is the width of the input feature map. $x_{c}(h,j)$ is the input along the horizontal direction of the feature map, $x_{c}(i,w)$ is the input along the vertical direction of the feature map. […] denotes the concatenation operation along spatial dimensions. δ is the nonlinear activation function. σ is the sigmoid function. $y_{n}(i,j)$ represents the output of the (*n*)-th channel, and $x_{n}(i,j)$ represents the input to the (*n*)-th channel. $g_{n}^{h}(i,j)$ and $g_{n}^{w}(i,j)$ represent the weights of the (*n*)-th channel along the horizontal and vertical directions, respectively.

**Figure 6:**
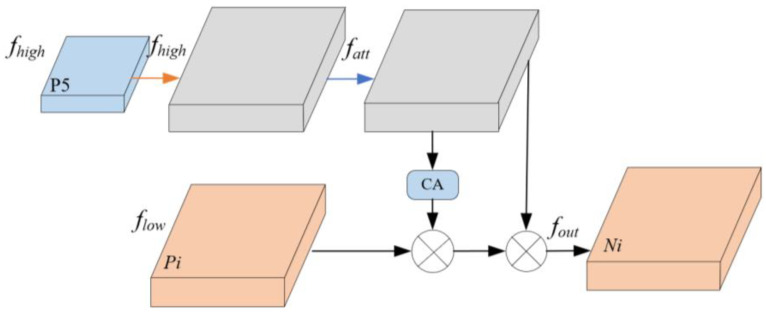
SFF network structure.

In the feature fusion module, to mitigate the structural conflict where high-level features lack precise localization ability and low-level features lack semantic expression ability, HSFPN further optimizes the SFF mechanism, as shown in Figure 6. This mechanism uses high-level features as guidance weights to filter and emphasize key semantic information embedded in low-level features. This fusion process achieves a balance between semantic richness and spatial accuracy. Finally, the filtered low-level features are effectively fused with high-level semantic features, significantly enhancing the model’s feature representation ability. The feature selection fusion process is expressed in Formulas (9) and (10):

(9) $$f_{att}=\mathrm{BL}(T-\mathrm{Conv}(f_{high}))$$

(10) $$f_{out}=f_{low}*\mathrm{CA}(f_{att})+f_{att}$$

where $f_{att}$ represents the new features obtained after sampling, BL represents bilinear interpolation, T-Conv () denotes transposed convolution, $f_{high}$ and $f_{low}$ are the high and low-scale features, $f_{out}$ represents the feature obtained after fusion of low and high-scale features.

#### 2.2.3. Detect_LIH Lightweight Detection Head

**Figure 7:**
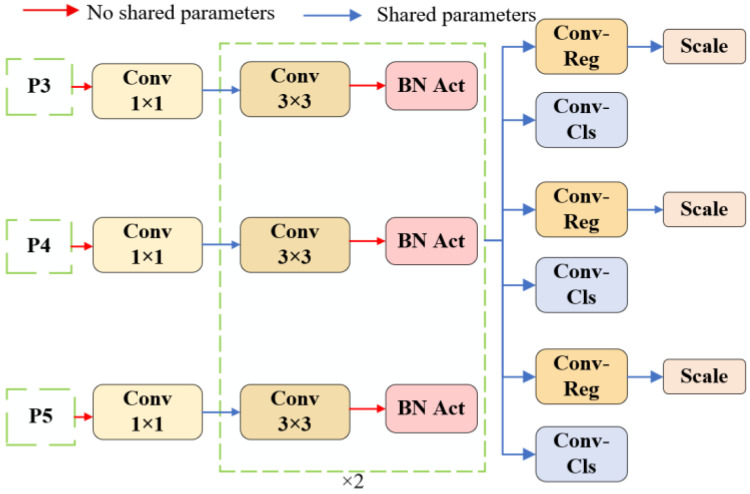
Detect_LIH network structure.

The detection head in YOLOv8n consists of multiple convolutional layers, each performing independent convolution operations, which significantly increases the model’s parameter count and leads to redundancy in the model structure and waste of computational resources. To effectively reduce the memory footprint of the model, this study proposes a lightweight design approach and optimizes the YOLOv8n model’s detection head, resulting in a lightweight shared convolution detection head called Detect_LIH, as shown in Figure 7.

In the Detect_LIH detection head, P3, P4, and P5 correspond to the prediction branches for small, medium, and large-scale targets, respectively. Each branch first performs feature extraction through a 1 × 1 convolutional layer. Given that feature maps from different scale branches exhibit significant differences in numerical distribution, layer normalization operations based on mean and variance are introduced into each branch to enhance training stability and convergence speed. Subsequently, the parameters from the 1 × 1 convolution are shared with the subsequent 3 × 3 convolution layers to form a shared parameter module. This module consists of 3 × 3 convolution layers and batch normalization (BN) layers to efficiently extract discriminative features and share core feature extraction parameters with convolution regularization layers (Conv-Reg) and loss function modules, as shown in Formula (11). Due to the scale differences in detection targets, the feature map, after passing through Conv-Reg, is scaled using the scale layer to further enhance multi-scale target detection adaptability and prediction accuracy. The scale layer dynamically adjusts the feature magnitude, as shown in Formulas (12) and (13), while reducing the model’s parameter count and computational overhead, effectively mitigating detection accuracy loss:

(11) $$g_{out}=f_{w}(g_{in})$$

(12) $$X_{scale,i}=F.view(X_{initial},scale\_factors=s)$$

(13) $$X_{combined}=concat(X_{scale1},X_{scale2},X_{scale3}\cdot \cdot \cdot )$$

where $g_{out}$ represents the output feature, $g_{in}$ represents the input feature, $f_{w}(\cdot )$ represents feature extraction, $X_{scale,i}$ represents the scaled tensor, *S* is the scaling factor, F.view represents a function for adjusting the tensor size, $X_{initial}$ represents the input initial tensor, $X_{combined}$ represents the result of scaling and concatenating multiple tensors.

#### 2.2.4. Loss Function Optimization and Improvement

The default loss function in the YOLOv8 base model is CIoU, which degrades to IoU loss when the aspect ratio of the predicted bounding box and the ground truth box is linear. This causes anchor boxes to expand during the regression process, significantly affecting the convergence speed and failing to fully account for the differences between bounding boxes. To address the detection accuracy loss and slow convergence caused by this defect, this study proposed an optimization method, PIoUv2 [26], to improve the base model’s loss function. This method accelerates the model’s convergence and enhances detection accuracy, with the computational expressions shown in Formulas (14)–(19):

(14) $$f(x)=1-e^{-x^{2}}$$

(15) PIoU=IoU−f(P),−1≤PIoU≤1

(16) $$L_{PIoU}=1-PIoU=L_{IoU}+f(P),0\leq L_{PIoU}\leq 2$$

(17) $$q=e^{-p},q\in (0,1]$$

(18) $$u(x)=3x\cdot e^{-x^{2}}$$

(19) $$L_{PIoU_{-v2}}=u(\lambda q)\cdot L_{PIoU}=3\cdot (\lambda q)\cdot e^{-{\left(\lambda q\right)}^{2}}\cdot L_{PIoU}$$

In these formulas, u(λq) represents the attention function. λ represents hyperparameters. q is the penalty factor in PIoU. When q=1, P=0, it indicates that the predicted bounding box perfectly overlaps with the ground truth bounding box. The newly introduced attention mechanism allows the model to focus more effectively on medium-quality anchor boxes, reducing the negative impact of low-quality anchor boxes on the gradients.

### 2.3. Performance Evaluation Metrics

This study primarily provides a lightweight and real-time visual detection model for hawthorn harvesting robots in complex orchard environments, aiming to reduce the model’s memory usage and parameter computational load while ensuring accuracy. Therefore, the performance evaluation metrics selected for this study include mean average precision (mAP), precision (P), recall (R), model size, number of parameters, and computational load.

### 2.4. Environment Configuration and Training Strategy

**Figure 8:**
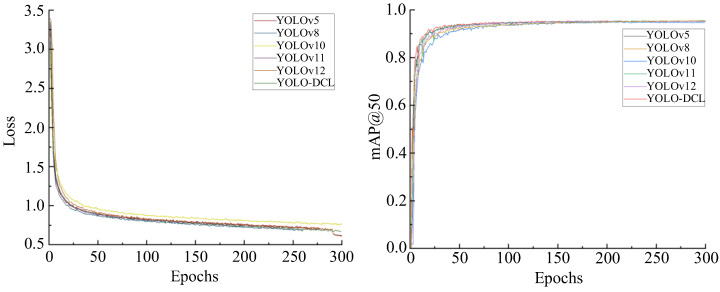
Loss and mAP@50 curves.

The model training in this study was performed on a Windows 11 Professional system with the following hardware and software configuration: an AMD Ryzen Threadripper PRO 3975WX 32-core processor at 3.50 GHz, 384 GB of RAM, an NVIDIA RTX A5000 GPU with 24 GB of VRAM, and corresponding software such as Anaconda 3.8, Python 3.8, OpenCV, CUDA 11.7, and PyTorch 1.13. In the model training setup, the mosaic data augmentation method was employed, using the SGD optimizer with a momentum factor of 0.937, an initial learning rate of 0.01, a weight decay coefficient of 0.005, a batch size of 16, and 300 epochs. The training process of the models is shown in Figure 8. Compared to other models, the improved YOLO-DCL model converges faster in terms of loss, demonstrating high learning efficiency. When the number of iterations reaches around 270, the model converges and stabilizes around 0.61, with an mAP@50 value of 95.6 and a model size of 2.46 MB.

## 3. Results and Analysis

### 3.1. Performance Comparison of Different Feature Extraction Networks

To assess the performance advantages of the proposed DGCST feature extraction network on the complex orchard hawthorn dataset, this study conducted a comparative experiment with several recently popular feature extraction networks, such as ConvNextV2 [27], EfficientViT [28], FasterNet [29], MobileNetV4 [30], LSKNet [31], and GhostNet [32]. The experimental results are detailed in Table 1.

As shown in the experimental results of Table 1, DGCST outperforms in several dimensions, including model memory usage, parameter size, and computational complexity in the complex orchard hawthorn dataset. While maintaining a mean average precision (mAP@50) of 95.3%, DGCST reduces the parameter count, computation load, and model size by 26.7%, 23.2%, and 25.2%, respectively, compared to the baseline model YOLOv8n. Compared to ConvNextV2, although DGCST’s mAP@50 is slightly lower by 0.2 percentage points, it demonstrates significant advantages in model lightweighting, with a 61.4%, 55.3%, and 59.5% reduction in parameter count, computation load, and model size, respectively. Additionally, when compared to EfficientViT, FasterNet, MobileNetV4, LSKNet, and GhostNet, DGCST shows better performance in terms of hawthorn target detection accuracy while achieving significant compression in model size: parameter counts decreased by 45.0%, 47.6%, 61.4%, 62.7%, and 4.5%, respectively; computational load dropped by 33.7%, 41.1%, 72.1%, 68.2%, and 0%; and model size reduced by 46.5%, 45.7%, 59.9%, 61.3%, and 4.3%, respectively. In summary, DGCST not only excels in small target detection accuracy for hawthorn but also shows a clear advantage in model lightweighting, indicating its promising application prospects in resource-constrained environments.

### 3.2. Ablation Study

To verify the efficacy of the four proposed enhancements (DGCST, HSFPN, Detect_LIH, PIoUv2), ablation experiments were conducted using the YOLOv8n model as the baseline. Each enhancement was incorporated sequentially at its designated location: DGCST in the backbone, HSFPN in the neck module, Detect_LIH in the detection head, and PIoUv2 replacing the bounding box loss. Results are presented in Table 2.

Based on the experimental results in Table 2, compared to the baseline model YOLOv8n, the optimization of the backbone network with the DGCST dynamic group shuffle convolution module leads to a 26.7%, 23.2%, and 25.2% reduction in parameter count, computational load, and model size, respectively, while maintaining the same mAP@50. This significantly reduces the model’s complexity, demonstrating that the YOLOv8n-DGCST backbone structure offers better computational efficiency and deployment adaptability, making it suitable for use in complex orchard harvesting robot vision systems.

Subsequently, the improved HSFPN feature fusion module was introduced based on YOLOv8n-DGCST. The experiment showed that this module increased mAP@50 by 0.2 percentage points to 95.5%, while reducing parameter count, computation load, and model size by 31.8%, 6.3%, and 29.0%, respectively. The performance improvement was attributed to the multi-scale feature selection and fusion strategy introduced by HSFPN, which enhanced detection accuracy while effectively reducing redundant computations.

Further optimization of the detection head structure resulted in a 20.0%, 18.6%, and 22.2% reduction in parameter count, computational load, and model size, respectively, while maintaining the same mAP@50. The results indicate that this detection head optimization significantly improves model inference efficiency and embedded deployment capability.

Finally, the introduction of the improved loss function further enhanced model performance. With the network structure and computational load unchanged, mAP@50 increased to 95.6%. By integrating all of the aforementioned optimizations, the model was named YOLO-DCL. Comprehensive analysis shows that YOLO-DCL improves detection accuracy by 0.3 percentage points compared to the baseline model YOLOv8n, while reducing parameter count by 60.0%, computational load by 41.5%, and model size by 58.7%. These results fully demonstrate that YOLO-DCL achieves a better accuracy–efficiency balance, providing an effective solution for target detection tasks in orchard hawthorn harvesting robots on low-computational platforms.

### 3.3. Performance Comparison with Mainstream Models

To further verify the detection performance of the proposed YOLO-DCL model on hawthorn small targets, we conducted a comparative experiment with several mainstream models, including Fast R-CNN, SSD, YOLOv5, YOLOv7, YOLOv8, YOLOv10, YOLOv11, and YOLOv12. The experimental results are shown in Table 3.

Based on the experimental data in Table 3, YOLO-DCL exhibits significant advantages in parameter count, computational load, and model size compared to Fast R-CNN, SSD, YOLOv5, YOLOv7, YOLOv8, YOLOv10, YOLOv11, and YOLOv12 baseline models. Compared to the original improved YOLOv8n model, YOLO-DCL reduces parameter count, computational load, and model size by 60.0%, 41.5%, and 58.7%, respectively. Compared to the latest YOLOv12 model, these reductions are 52.0%, 26.2%, and 53.1%, respectively. Additionally, in terms of mean average precision (mAP@0.5) and recall (R), the proposed model performs best, achieving mAP@0.5 of 95.6% and recall of 90.1%. While YOLO-DCL’s precision (P) is slightly lower than that of other YOLO series models, it still achieves a precision of 91.6%, which is sufficient to meet the detection needs of hawthorn harvesting robots in complex orchard environments. Notably, YOLO-DCL has a clear advantage in lightweight design, enabling it to operate efficiently on embedded low-computational devices. Therefore, YOLO-DCL has high deployment potential in orchard harvesting robots and similar applications.

### 3.4. Performance Comparison in Different Orchard Scenarios

**Figure 9:**
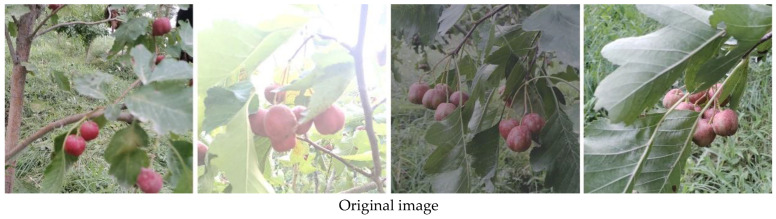

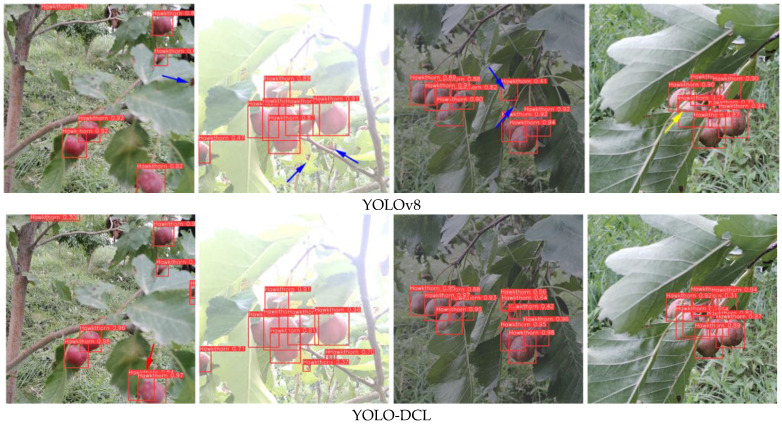
Detection performance comparison in different scenarios.

To systematically evaluate the real-world detection performance of the proposed YOLO-DCL model in complex orchard environments, we designed and conducted comparative experiments with the mainstream YOLOv8 model as the baseline. The experiments were based on a unified test set, covering four representative complex scenarios in orchards: wide-angle, backlight, cloudy, and target occlusion. As shown in Figure 9, blue arrows indicate missed targets, red arrows indicate overlapping detection areas, and yellow arrows indicate incomplete coverage of targets.

From the results in Figure 9, it can be observed that in the wide-angle scenario, YOLOv8 exhibited significant missed detections (indicated by the blue arrows), with overall detection confidence lower. In contrast, YOLO-DCL effectively detected more hawthorn targets and showed better detection confidence. However, YOLO-DCL experienced some repeated detection issues in certain images (as indicated by the red arrows), suggesting room for further optimization. In the backlight and cloudy conditions, YOLOv8 showed poor detection ability for small hawthorn targets, resulting in multiple missed detections. In contrast, YOLO-DCL demonstrated stronger robustness in these scenarios, successfully detecting all targets with significantly higher confidence, indicating its superior light invariance and small target detection capability. In the target occlusion scenario, YOLOv8’s predicted bounding boxes often failed to fully cover the occluded fruits (as indicated by yellow arrows), leading to incomplete detection information. In contrast, YOLO-DCL provided complete predictions for occluded targets, demonstrating higher spatial perception ability and boundary regression accuracy.

In summary, although YOLO-DCL exhibited some instances of repeated detections, it still achieved significant reductions in parameter count, computational load, and model size, while maintaining or even surpassing the detection performance of the original model. Especially in complex scenarios, YOLO-DCL demonstrated superior mAP@0.5 and recall, showcasing excellent stability and generalization ability in target detection. Moreover, considering the practical need for embedded systems with limited resources, YOLO-DCL, with its highly lightweight structure, has considerable deployment potential, particularly in low-power, real-time visual perception tasks such as orchard hawthorn harvesting robots. It holds significant engineering application value.

### 3.5. Edge Device Deployment and Validation

**Figure 10:**
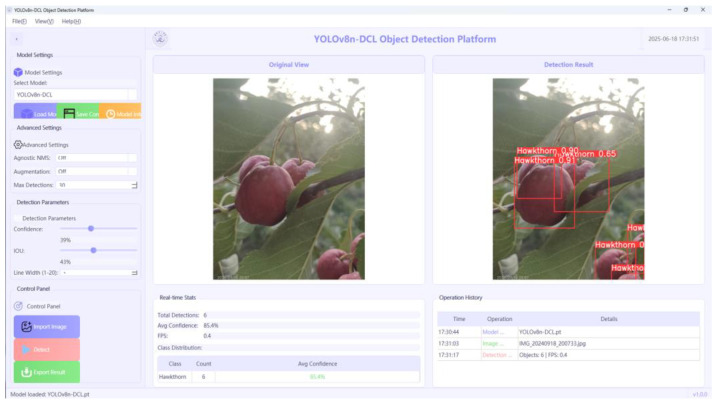
Visualization detection system.

**Figure 11:**
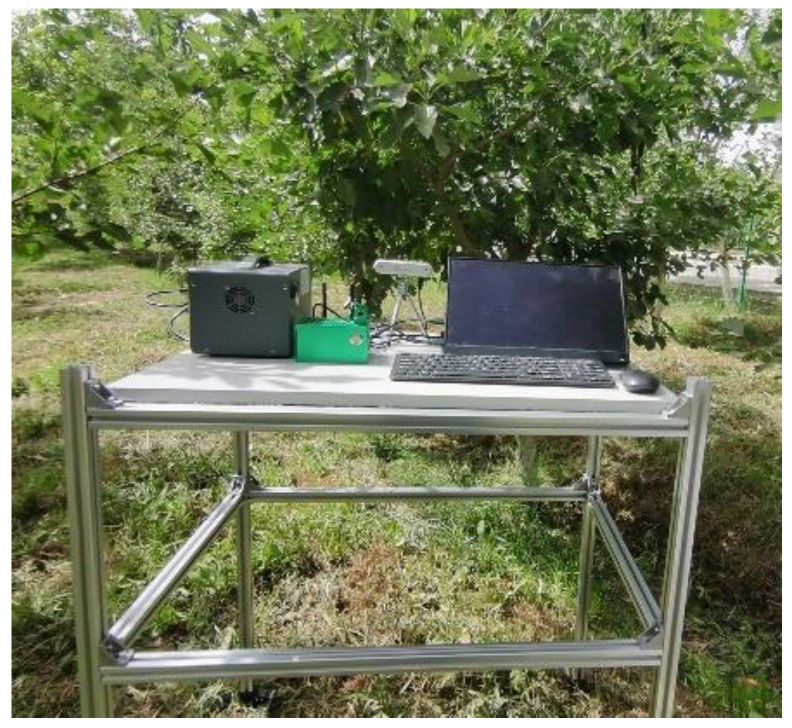
System deployment.

To intuitively evaluate the detection performance of the improved model in real-world applications, we designed and developed a hawthorn fruit detection and visualization system based on the PySide6 framework. The system architecture is illustrated in Figure 10. It supports real-time switching between different detection models and allows users to adjust the IoU threshold. Detected images are displayed in real-time on the right panel of the main interface, while detailed detection results—such as object categories, target counts, and average confidence scores—are shown at the bottom of the interface. These outputs help each image. Additionally, a historical information module on the left panel records user operation logs, deployed on the NVIDIA Jetson Xavier NX 16 GB device (NVIDIA Corporation, Santa Clara, CA, USA), which is equipped with 384 CUDA cores and 48 tensor cores, offering robust computational performance. To accelerate inference, the TensorRT framework was employed, and the overall deployment architecture is depicted in Figure 11.

According to the deployment test results, a comparative analysis of the detection performance on the NVIDIA Jetson Xavier NX 16GB device before and after the model improvement shows that the base YOLOv8 model achieved a frame rate of 23.8 FPS, while the improved YOLO-DCL model reached 31.5 FPS. The corresponding inference times were 43.5 ms and 25.6 ms, respectively. Specifically, YOLO-DCL achieved a 32.4% improvement in FPS and a 41.1% reduction in inference time. These results demonstrate that the improved YOLO-DCL model significantly outperforms the original baseline model on embedded devices with limited computational power, effectively meeting the real-time detection requirements of crop-picking robots, such as those used for hawthorn in smart agriculture applications.

## 4. Conclusions

To overcome challenges of missed detections, false alarms, and limited real-time performance in vision systems for hawthorn-picking robots operating under complex field conditions, this study proposes YOLO-DCL—a real-time detector based on the YOLOv8n architecture. The model incorporates lightweight convolutions and was rigorously validated through theoretical analysis and experimental deployment. The key findings are as follows:

(1) To enhance detection efficiency and real-time performance, YOLO-DCL incorporates several key architectural modifications. The baseline model’s C2f module in the backbone is replaced by a DGCST. The neck network is redesigned using an HSPFN, and Detect_LIH, is introduced. Furthermore, the loss function is updated from CIoU to PIoUv2. These enhancements enable YOLO-DCL to achieve a 0.3% improvement in detection accuracy compared to the baseline, while simultaneously reducing parameters by 60.0%, computational cost by 41.5%, and model size by 58.7%.
(2) To further validate YOLO-DCL’s efficacy for detecting small hawthorn targets, comparative evaluations were performed against prominent models: Fast R-CNN, SSD, YOLOv5, YOLOv7, YOLOv8, YOLOv10, YOLOv11, and YOLOv12. The results indicate that YOLO-DCL achieves superior overall performance, excelling in model complexity (parameters, computational load, size) and detection accuracy (mAP). This demonstrates its potential as an efficient solution for hawthorn detection in orchard environments using low-computation platforms.
(3) Experimental validation conducted on a Jetson Xavier platform demonstrated the efficacy of a real-time hawthorn detection system developed using the PySide6 framework. The proposed YOLO-DCL model exhibited a 32.4% increase in frames per second (FPS) and a 41.1% reduction in inference latency compared to the baseline. These performance gains confirm the model’s suitability for fulfilling the real-time operational requirements of robotic fruit harvesting systems.

However, the annotation strategy focusing solely on harvestable fruits may limit the model’s sensitivity to heavily occluded or unripe targets. While this approach streamlines training, it could lead to under-detection in scenarios where non-harvestable fruits partially obstruct harvestable ones or under varying maturity conditions. Future work will focus on integrating depth perception or 3D reconstruction techniques to provide spatial information about hawthorn fruits. This enhancement is expected to further improve detection robustness against occlusions and enable more precise localization for robotic picking operations in complex orchard environments. Furthermore, the coefficient of friction between the end-effector and the hawthorn surface significantly influences the grasping force control. In future studies, we plan to integrate the visual inspection results with the robotic manipulation control module and employ a nonlinear friction model [33] for analysis. This will facilitate the establishment of a database documenting the friction coefficients between the end-effector and hawthorn skin, enabling the refinement of grasping force control strategies.

## Figures and Tables

**Table 1:** Experimental comparison of different feature extraction networks.

Model	mAP@0.5/%	Params/M	FLOPs/G	Weights/MB
YOLOv8-C2f	95.3	3.0	8.2	5.95
YOLOv8-ConvNextV2	95.5	5.7	14.1	11.0
YOLOv8-EfficientViT	95.3	4.0	9.5	8.32
YOLOv8-FasterNet	95.3	4.2	10.7	8.2
YOLOv8-MobileNetV4	95.0	5.7	22.6	11.1
YOLOv8-LSKNet	94.4	5.9	19.8	11.5
YOLOv8-Ghost	95.0	2.3	6.3	4.65
YOLOv8-DGCST	95.3	2.2	6.3	4.45

**Table 2:** Ablation experiment results.

Model	DGCST	HSFPN	Detect_LIH	PIoUv2	Params/M	FLOPs/G	P/%	R/%	mAP@0.5/%	Weights/MB
YOLOv8n	×	×	×	×	3.0	8.2	91.8	89.9	95.3	5.95
	√	×	×	×	2.2	6.3	93.5	87.3	95.3	4.45
	√	√	×	×	1.5	5.9	91.0	89.8	95.5	3.16
	√	√	√	×	1.2	4.8	93.1	88.3	95.5	2.46
	√	√	√	√	1.2	4.8	91.6	90.1	95.6	2.46

**Table 3:** Performance comparison with mainstream models.

Model	Params/M	FLOPs/G	P/%	R/%	mAP@0.5/%	Weights/MB
Fast R-CNN	136.7	369.7	65.4	84.5	83.5	108
SSD	23.6	273.2	88.1	67.0	78.0	90.6
YOLOv5	2.5	7.2	92.6	88.7	95.4	5.02
YOLOv6	4.2	11.9	93.3	87.9	95.0	32.7
YOLOv7	6.0	13.2	91.7	90.5	95.3	11.6
YOLOv8	3.0	8.2	91.8	89.8	95.3	5.95
YOLOv10	2.7	8.4	91.8	88.2	94.8	5.48
YOLOv11	2.5	6.4	93.8	88.5	95.3	5.22
YOLOv12	2.5	6.5	92.7	87.5	95.3	5.25
YOLO-DCL	1.2	4.8	91.6	90.1	95.6	2.46

## Data Availability

The data in the paper is presented in the form of charts and graphs. For more detailed data, please obtain it from the corresponding author.

## References

[1] Dong J., Chen J., Gong S., Xu J., Xu X., Zhang T. (2021). Research progress on chemical constituents and pharmacological effects of crataegi fructus and predictive analysis on Q-Marker. Chin. Tradit. Herb. Drugs.

[2] Dong N., Wang Y., Zheng S., Wang S. (2022). The current situation and development suggestions of China’s hawthorn industry. China Fruits.

[3] Zhai Y. (2018). Structure Design and Motion Analysis of a Hawthorn Picking Machine. Master’s Thesis.

[4] Song H., Shang Y., He D. (2023). Review on deep learning technology for fruit target recognition. Trans. Chin. Soc. Agric. Mach..

[5] Ma M., Guo J. (2024). Research status of machine vision technology for fruit positioning. Chin. South. Agric. Mach..

[6] Mo S., Dong T., Zhao X., Kan J. (2022). Discriminant model of banana fruit maturity based on genetic algorithm and SVM. J. Fruit Sci..

[7] Zou W. (2023). Research on citrus fruit maturity grading based on machine vision technology. Agric. Technol..

[8] Dong G., Xie W., Huang X., Qiao Y., Mao Q. (2023). Review of small object detection algorithms based on deep learning. Comput. Eng. Appl..

[9] Girshick R. Fast R-CNN. Proceedings of the IEEE International Conference on Computer Vision.

[10] Liu W., Anguelov D., Erhan D., Szegedy C., Reed S., Fu C., Berg A. SSD: Single shot multibox detector. Proceedings of the Computer Vision-ECCV 2016: 14th European Conference.

[11] Redmon J., Divvala S., Girshick R., Farhadi A. You only look once: Unified, real-time object detection. Proceedings of the IEEE Conference on Computer Vision and Pattern Recognition.

[12] Li L., Liang J., Zhang Y., Zhang G., Chun C. (2024). Accurate detection and localization method of citrus targets in complex environments based on improved YOLOv5. Trans. Chin. Soc. Agric. Mach..

[13] Gu H., Li Z., Li T., Li T., Li N., Wei Z. (2024). Lightweight detection algorithm of seed potato eyes based on YOLOv5. Trans. Chin. Soc. Agric. Eng..

[14] Luo Z., He C., Chen D., Li P., Sun Q. (2024). Passion fruit rapid detection model based on lightweight YOLOv8s-GD. Trans. Chin. Soc. Agric. Mach..

[15] Dong G., Chen X., Fan X., Zhou J., Jiang H., Cui C. (2024). Detecting Xinmei fruit under complex environments using improved YOlOv5s. Trans. Chin. Soc. Agric. Eng..

[16] Song H., Wang Y., Wang Y., Lü S., Jiang M. (2022). Camellia oleifera fruit detection in natural scene based on YOLOv5s. Trans. Chin. Soc. Agric. Mach..

[17] Yang S., Wang W., Gao S., Deng Z. (2023). Strawberry ripeness detection based on YOLOv8 algorithm fused with LW-Swin Transformer. Comput. Electron. Agric..

[18] Wu F., Duan J., Ai P., Chen Z., Yang Z., Zou X. (2022). Rachis detection and three-dimensional localization of cut off point for vision-based banana robot. Comput. Electron. Agric..

[19] Zheng Z., Hu Y., Qiao Y., Hu X., Huang Y. (2022). Real-time detection of winter jujubes based on improved YOLOX-nano network. Remote Sens..

[20] Li S., Huang H., Meng X., Wang M., Li Y., Xie L. (2023). A glove-wearing detection algorithm based on improved YOLOv8. Sensors.

[21] Gong W. (2024). Lightweight Object Detection: A Study Based on YOLOv7 Integrated with ShuffleNetv2 and Vision Transformer. arXiv.

[22] Su Z., Fang L., Kang W., Hu D., Pietikäinen M., Liu L. Dynamic Group Convolution for Accelerating Convolutional Neural Networks. Proceedings of the 16th European Conference on Computer Vision (ECCV 2020).

[23] Huang Z., Ben Y., Luo G., Cheng P., Yu G., Fu B. (2021). Shuffle transformer: Rethinking spatial shuffle for vision transformer. arXiv.

[24] Hou Q., Zhou D., Feng J. Coordinate attention for efficient mobile network design. Proceedings of the 2021 IEEE Conference on Computer Vision and Pattern Recognition.

[25] Lin T.Y., Dollár P., Girshick R., He K., Hariharan B., Belongie S. Feature pyramid networks for object detection. Proceedings of the IEEE Conference on Computer Vision and Pattern Recognition.

[26] Liu C., Wang K., Li Q., Zhao F., Zhao K., Ma H. (2024). Powerful-IoU: More straightforward and faster bounding box regression loss with a nonmonotonic focusing mechanism. Neural Netw..

[27] Woo S., Debnath S., Hu R., Chen X., Liu Z., Kweon I.S., Xie S. Convnext v2: Co-designing and scaling convnets with masked autoencoders. Proceedings of the IEEE/CVF Conference on Computer Vision and Pattern Recognition.

[28] Liu X., Peng H., Zheng N., Yang Y., Hu H., Yuan Y. Efficientvit: Memory efficient vision transformer with cascaded group attention. Proceedings of the IEEE/CVF Conference on Computer Vision and Pattern Recognition.

[29] Chen J., Kao S.H., He H., Zhuo W., Wen S., Lee C.H., Chan S.H.G. Run, don’t walk: Chasing higher FLOPS for faster neural networks. Proceedings of the IEEE/CVF Conference on Computer Vision and Pattern Recognition.

[30] Qin D., Leichner C., Delakis M., Fornoni M., Luo S., Yang F., Wang W., Banbury C., Ye C., Akin B. MobileNetV4: Universal models for the mobile ecosystem. Proceedings of the European Conference on Computer Vision.

[31] Li Y., Hou Q., Zheng Z., Cheng M.M., Yang J., Li X. Large selective kernel network for remote sensing object detection. Proceedings of the IEEE/CVF International Conference on Computer Vision.

[32] Han K., Wang Y., Tian Q., Guo J., Xu C., Xu C. Ghostnet: More features from cheap operations. Proceedings of the IEEE/CVF Conference on Computer Vision and Pattern Recognition.

[33] Fotuhi M.J., Hazem Z.B., Bingül Z. (2018). Adaptive joint friction estimation model for laboratory 2 DOF double dual twin rotor aerodynamical helicopter system. Proceedings of the 2018 6th International Conference on Control Engineering & Information Technology (CEIT).

